# Symptomatic Menopausal Transition Increases the Risk of New-Onset Depressive Disorder in Later Life: A Nationwide Prospective Cohort Study in Taiwan

**DOI:** 10.1371/journal.pone.0059899

**Published:** 2013-03-27

**Authors:** Mu-Hong Chen, Tung-Ping Su, Cheng-Ta Li, Wen-Han Chang, Tzeng-Ji Chen, Ya-Mei Bai

**Affiliations:** 1 Department of Psychiatry, Taipei Veterans General Hospital, Taipei, Taiwan; 2 Department of Psychiatry, College of Medicine, National Yang-Ming University, Taipei, Taiwan; 3 Department of Family Medicine, Taipei Veterans General Hospital, Taipei, Taiwan; 4 Institute of Brain Science, National Yang-Ming University, Taipei, Taiwan; 5 Institute of Hospital and Health Care Administration, National Yang-Ming University, Taipei, Taiwan; University of Washington, United States of America

## Abstract

**Introduction:**

The role of the menopausal transition and associated menopausal symptoms in the occurrence of depressive disorders has been discussed and debated for a long time. Most previous clinical studies had limited case samples, and did not control the attributable risk of medical comorbidities.

**Methods:**

Patients with a diagnosis of symptomatic menopausal transition and without a psychiatric history were enrolled in 2000 in Taiwan, and compared with age-matched controls (1∶4). These subjects were followed to the end of 2010 to investigate the association between symptomatic menopausal transition and new-onset depressive disorder; the effect of medical comorbidities was also assessed.

**Results:**

A total of 5,837 women with symptomatic menopausal transition were identified, and compared with 23,348 age-matched controls in 2000. The follow-up showed that symptomatic menopausal transition was an independent risk factor for major depression (hazard ratio[HR]: 2.18, 95%CI: 1.79∼2.65) and any depressive disorder (HR: 2.34, 95%CI: 2.08∼2.63) after adjusting age at enrollment, monthly income, residence location, level of urbanization, and comorbid medical diseases. In addition, medical comorbidities, including cerebrovascular disease (HR: 1.77, 95% CI: 1.52∼2.07), cardiovascular diseases (HR: 1.35, 95% CI: 1.15∼1.57), congestive heart failure (HR: 1.35, 95% CI: 1.04∼1.75), and liver diseases (HR: 1.19, 95% CI: 1.03∼1.36) increased the risk of developing any depressive disorder.

**Conclusion:**

Our population cohort study, with the largest study sample and medical record diagnosis thus far, supports an association between symptomatic menopausal transition and depressive disorder in midlife women, and an increased risk of depressive disorder with medical comorbidities.

## Introduction

Menopause is a transitional phase, from a reproductive state to a non-reproductive state due to ovarian function decline and hyperactive change in the hypothalamus-pituitary-gonadal axis, including increased secretion of follicle-stimulating hormone (FSH) and luteinizing hormone (LH), typically occurring in midlife in women [1]–[2]. This climacteric process coincides with a wide range of physical and mental symptoms [1]–[2]. Symptomatic menopausal transition manifests many different somatic symptoms, including vasomotor symptoms (i.e., hot flushes and cold or night sweats), sexual discomfort, vaginal dryness, headaches, and joint pains [3]–[4]. In addition, symptomatic menopausal transition includes psychiatric symptoms, such as depressive and anxious mood, trouble sleeping, feelings of panic, and irritability [3]–[4]. In general, in clinical practice, the diagnosis of symptomatic menopausal transition focused more on these somatic symptoms instead of psychiatric symptoms [2], [5].

The association between menopausal transition and depression in later life has become an object of public and scientific concern in recent years. The Harvard Study of Moods and Cycles, which involved 460 women aged 36 to 45 years, and a 6-year follow-up using telephone interviews and self-administered questionnaires, demonstrated a relationship between the menopausal transition and the onset of first lifetime episode of depression [6]. The perimenopause (menopausal transition) was defined as an absolute change of 7 days or greater in menstrual cycle length and an obvious change in menstrual flow amount or duration 6. Cohen et al reported that women without a history of mood disorder who entered the menopausal transition were twice as likely to develop significant depressive symptoms as those who remained premenopausal (odds ratio [OR] of 1.8 and 95% confidence interval [CI] of 1.0–3.2), and that women with symptomatic menopausal transition had a greater risk of depression (OR: 2.2, 95%CI: 1.1–4.2) [6]. Bromberger et al followed 266 midlife women who were pre-menopausal at entry into the Study of Women's Health Across the Nation (SWAN) for 7 years, and found that symptomatic menopausal transition with frequent vasomotor symptoms was a significant predictor of new-onset major depression (HR: 2.14, 95%CI: 1.07∼4.30) [7]. Bosworth et al assessed 581 women aged 45 to 54 years, and suggested that climacteric symptoms but not menopausal status were associated with higher rates of depressive symptoms [8]. Analyzing the data from the nationwide 2002 Health Promotion Knowledge, Attitude, and Performance Survey in Taiwan, Lin et al suggested that perimenopausal status was significantly associated with depressive symptoms (OR: 1.97, 95%CI: 1.24∼3.14) [9]. But the relationship between menopausal transition and depression is still debated. Previous studies reported that in the premenopause period, the prevalence rates of major depression ranged from 5.8% to 11%; in the perimenopause period, from <4% to 9.1%; and in the postmenopause stage, from <1% to 9.8% [10]–[12]. The prevalence rate of major depression seemed not to differ significantly during these three periods. In 2005, the US National Institutes of Health claimed that because of the multiple potential causes of mood changes (i.e., history of prior depression, life stress, and general health) and the relatively high proportion of women reporting 1 or more affective symptoms, it is difficult to establish whether menopause causes any increase in the prevalence of mood symptoms during the perimenopausal years [13]. They concluded that the data on the association between menopause and depression were mixed, and that there is limited evidence that ovarian changes associated with menopause might be a cause of depression, anxiety, and irritability [13]. However, these mixed and inconsistent results may be due to methodological limitations, including the use of self-report surveys and different definitions of depression. Another possible confounding factor is that the menopausal transition increases the comorbidity of many medical illnesses, including cerebrovascular disease, coronary artery disease, and diabetes mellitus [1], [14]–[24]. The aforementioned medical comorbidities are also regarded as risk factors for depressive symptoms/disorders [1], [14]–[24]. But the attributable risk of medical comorbidities for depression during the menopausal transition is rarely discussed. Other limitations of previous studies included limited sample sizes, the duration of follow-up, and the validity of depression diagnoses by self-report questionnaires.

In the present study, we used the Taiwan National Health Insurance Research Database (NHIRD), one of the biggest population-based medical databases in the world, and focused on the symptomatic menopausal transition, but not on the stages of the menopausal transition. We investigated the temporal relationship of symptomatic menopausal transition with new-onset depressive disorder. We hypothesized that symptomatic menopausal transition is an independent risk factor for major depression and any depressive disorder after adjusting for the medical comorbidities.

## Methods

### Data Source

The National Health Insurance (NHI) program was implemented in Taiwan in 1995, and offers universal health coverage for all its citizens. Since 2001, Taiwan’s NHI has covered 96.9% of all 23,000,000 residents of Taiwan. All citizens in Taiwan can seek medical help from this system. The National Health Research Institutes has provided a database of medical claims of 1,000,000 random subjects, approximately 4.3% of the population, for use in health service studies. This study used data from the Taiwan NHIRD covering the years 1996 to 2010. The data set includes all demographic and medical information on insured residents, including age, gender, residence location, prescription drugs, prescription date, and the disease diagnosis. The International Classification of Diseases, 9th Revision, Clinical Modification (ICD-9-CM) was used for the diagnosis. The completeness and accuracy of the NHIRD has been affirmed by the Department of Health and the Bureau of National Health Insurance through audit [25]. The NHIRD has been used extensively in many epidemiologic studies in Taiwan [26]–[27].

### Inclusion Criteria for Women with Symptomatic Menopausal Transition and the Control Cohort

This study consisted of a study cohort and a control cohort. The study cohort included all women with the diagnosis of symptomatic menopausal transition (ICD-9-CM code: 627.2) given by gynecologists between January 2000 and December 2000. The age at the diagnosis of symptomatic menopausal transition was defined as the age at enrollment. Those who were identified as having mental disorders (ICD-9-CM codes: 290∼319) during the preceding 4-year period (1996∼1999) were excluded. In addition, patients aged less than 18 years were eliminated, so as to limit our study sample to the adult population. Ultimately, 5837 women who had been diagnosed with symptomatic menopausal transition and had no history of mental disorder were included in our study cohort. The age- matched control cohort (4 for every patient in the study cohort) was randomly identified based on the subjects after eliminating patients who had been given a diagnosis of symptomatic menopausal transition anytime during the 10 year study period, and who had been diagnosed with a psychiatric disorder before the enrollment time. Diagnoses of major depression (ICD-9-CM codes: 296.2X and 296.3X) and any depressive disorder (ICD-9-CM codes: 296.2X, 296.3X, 300.4, and 311) given by board-certificated psychiatrists were identified during the follow-up duration (2000∼2010). All diagnoses were given at least twice by corresponding physicians for diagnostic validity. Information on medical comorbidities, residence location, level of urbanization, and monthly income was included for analysis. Medical comorbidities, as the confounding factors, were selected based on the Charlson comorbidity index, and included diabetes mellitus, congestive heart failure, chronic renal diseases (chronic glomerulonephritis, nephritis, nephropathy, chronic kidney disease, and renal failure), liver diseases (liver cirrhosis, chronic hepatitis, chronic liver disease), rheumatological diseases (systemic lupus erythematosus, systemic sclerosis, polymyositis, rheumatoid arthritis, rheumatoid lung, polymyalgia rheumatica), cerebrovascular diseases, and coronary artery diseases [28]–[29].

### Statistical Analysis

For cross-cohort comparison, the independent t test was used for continuous variables and Pearson's X^2^ test or Fisher's exact test for nominal variables, where appropriate. Multivariable Cox regression analysis was performed to evaluate the risk of hazard ratios (HR) with 95% CI of major depression and any depressive disorder with the symptomatic menopausal transition after adjusting for age at enrollment, gender, residence location, level of urbanization, monthly income, and comorbid medical diseases. In the Cox regression model, the development of major depression and any depressive disorder was defined as the dependent variable, and the major factor (symptomatic menopausal transition) and the various confounding factors (demographic data and medical co-morbidities during the whole follow-up) were defined as the independent variables. In order to test the proportional hazard assumption, log-minus-log plot was performed, and the proportional hazards assumption was not violated in both Cox regression models of major depression and any depressive disorder. A two-tailed *P*-value of less than 0.05 was considered statistically significant. All data processing and statistical analyses were performed with the Statistical Package for Social Sciences (SPSS) version 17 software (SPSS Inc) and Statistical Analysis Software (SAS) version 9.1 (SAS Institute, Cary, NC).

## Results

A total of 5837 women with symptomatic menopausal transition were identified, and compared with 23,348 age-matched controls in 2000. Regarding the demographic data, the women with symptomatic menopausal transition significantly resided less often in Northern Taiwan and more often in urbanized regions, and had the higher monthly income than those without symptomatic menopausal transition. Women with symptomatic menopausal transition had a higher incidence of developing major depression (2.8% vs. 1.2%, p<0.001) and any depressive disorder (7.8% vs. 3.1%, p<0.001) during the follow-up period. Moreover, a higher prevalence of comorbid medical illnesses at enrollment time and during the whole follow-up period was noted in women with symptomatic menopausal transition than in the control group, including diabetes mellitus (7.2% vs. 5.5%, p<0.001; 26.4% vs. 22.7%, p<0.001), chronic renal diseases (3.0% vs. 1.5%, p<0.001; 9.4% vs. 6.3%, p<0.001), liver diseases (9.3% vs. 5.0%, p<0.001; 27.6% vs. 17.6%, p<0.001), rheumatological diseases (1.5% vs. 0.7%, p<0.001; 4.0% vs. 2.0%, p<0.001), cerebrovascular diseases (0% vs. 0%; 14.9% vs. 9.7%, p<0.001), and coronary artery disease (3.9% vs. 2.2%, p<0.001; 17.1% vs. 11.5%, p<0.001). The aforementioned results must be interpreted with caution because they were uncorrected for confounding by age (Table 1 and Table 2).

**Table 1:** Distribution of characteristics of women with symptomatic menopausal transition and the control group.

	Women with symptomatic menopausaltransition (n = 5837)	Control cohort(n = 23,348)	*p* value
Age (year, SD)	63.98 (8.15)	63.98 (8.15)	
Gender (Female, n, %)	5837 (100)	23348 (100)	
Age at enrollment (year, SD)	52.05 (8.12)	52.05 (8.12)	
Major depression (n, %)	161 (2.8)	274 (1.2)	**<0.001**
Age at major depression (years)	57.26 (8.22)	57.96 (9.32)	0.431
Any depressive disorder (n, %)	457 (7.8)	729 (3.1)	**<0.001**
Age at any depressive disorder (years)	57.25 (8.47)	58.26 (9.44)	0.062
Residence location (n, %)			**<0.001**
Northern	2611 (44.7)	11306 (48.4)	
Middle	1063 (18.2)	3910 (16.7)	
Southern	1858 (31.8)	7217 (30.9)	
Eastern	305 (5.2)	915 (3.9)	
Level of urbanization (n, %)			**<0.001**
1 (most urban)	1906 (32.7)	7118 (30.5)	
2	1794 (30.7)	7035 (30.1)	
3	834 (14.3)	3518 (15.1)	
4	845 (14.5)	3255 (13.9)	
5 (most rural)	458 (7.8)	2422 (10.4)	
Income-related insured amount			**0.001**
<20,000NTD/month	3106 (53.2)	12585 (53.9)	
20,000∼39,999NTD/month	2162 (37.0)	8844 (37.9)	
≥40,000NTD/month	569 (9.7)	1919 (8.2)	

Major depression: 296.2X and 296.3X.

Any depressive disorder: 296.2X, 296.3X, 300.4, and 311.

**Table 2:** Medical comorbidity at enrollment and during the whole follow-up period among women with symptomatic menopausal transition (n = 5837) and the control group (n = 23,348).

	Women with symptomatic menopausaltransition (n = 5837)	Control cohort(n = 23,348)	*p* value
Medical comorbidities at enrollment (n, %)			
Diabetes mellitus	420 (7.2)	1277 (5.5)	<0.001
Congestive heart failure	49 (0.8)	123 (0.5)	0.006
Chronic renal diseases	176 (3.0)	357 (1.5)	<0.001
Liver diseases	545 (9.3)	1161 (5.0)	<0.001
Rheumatological diseases	88 (1.5)	158 (0.7)	<0.001
Cerebrovascular diseases	0 (0)	0 (0)	–
Coronary artery diseases	228 (3.9)	509 (2.2)	<0.001
Medical comorbidities during the whole follow-up period (n, %)			
Diabetes mellitus	1543 (26.4)	5295 (22.7)	<0.001
Congestive heart failure	203 (3.5)	798 (3.4)	0.812
Chronic renal diseases	550 (9.4)	1476 (6.3)	<0.001
Liver diseases	1609 (27.6)	4106 (17.6)	<0.001
Rheumatological diseases	234 (4.0)	470 (2.0)	<0.001
Cerebrovascular diseases	867 (14.9)	2263 (9.7)	<0.001
Coronary artery diseases	1001 (17.1)	2686 (11.5)	<0.001

**Figure 1:**
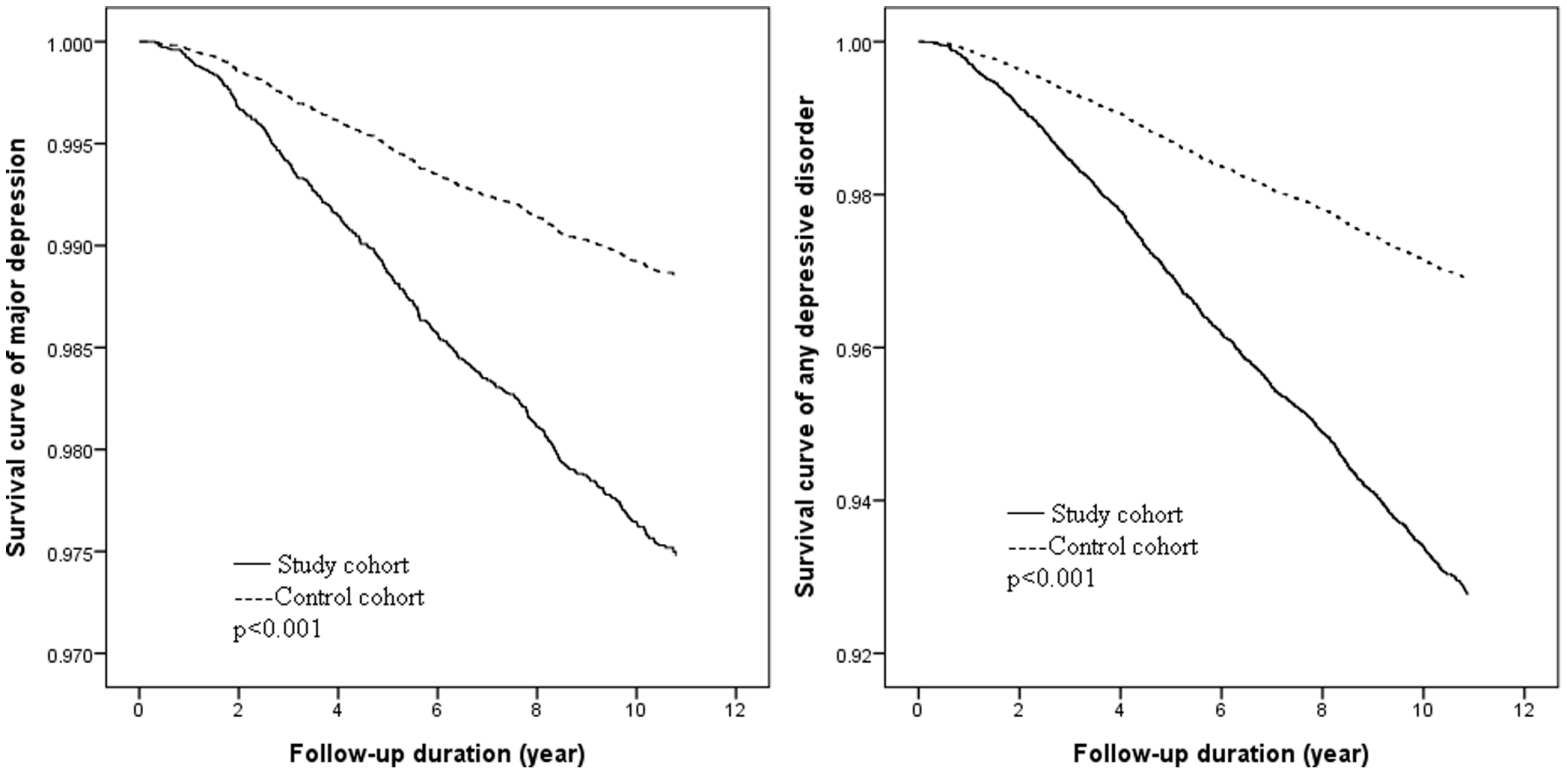
Survival curves of those with major depression or any depressive disorder among women with symptomatic menopausal transition and the control group.

Using multivariable Cox regression analysis, the follow-up showed that symptomatic menopausal transition was an independent risk factor for major depression (HR: 2.18, 95% CI: 1.79∼2.65) and any depressive disorder (HR: 2.34, 95% CI: 2.08∼2.63) after adjusting for age at enrollment, monthly income, residence location, level of urbanization, and comorbid medical diseases (Table 3); this was also demonstrated in the Kaplan-Meier survival curves (Figure 1). For the medical comorbidities, cerebrovascular diseases (HR: 1.77, 95% CI: 1.52∼2.07), coronary artery diseases (HR: 1.35, 95% CI: 1.15∼1.57), congestive heart failure (HR: 1.35, 95% CI: 1.04∼1.75), and liver diseases (HR: 1.19, 95% CI: 1.03∼1.36) also increased the risk of developing any depressive disorder (Table 3). In addition, older age at diagnosis of symptomatic menopausal transition presented a decreased risk of developing any depressive disorder but not major depression (HR: 0.99, 95% CI: 0.98∼1.00) (Table 3).

**Table 3:** Risk of developing major depression and any depressive disorder among women with symptomatic menopausal transition (n = 5837) compared to the control group (n = 23,348).

	Major depression			Any depressive disorder		
	HR	95%CI	*p*	HR	95%CI	*p*
Symptomatic menopausal transition	2.18	1.79∼2.65	**<0.001**	2.34	2.08∼2.63	**<0.001**
Age at enrollment	0.99	0.98∼1.00	0.057	0.99	0.98∼1.00	**0.006**
Medical comorbidities during the whole follow-up period						
Diabetes mellitus	1.13	0.90∼1.41	0.289	1.11	0.97∼1.27	0.125
Congestive heart failure	1.27	0.82∼1.98	0.285	1.35	1.04∼1.75	**0.026**
Chronic renal diseases	1.18	0.86∼1.64	0.311	1.16	0.96∼1.41	0.132
Liver diseases	1.18	0.94∼1.47	0.163	1.19	1.03∼1.36	**0.015**
Rheumatological diseases	1.04	0.607∼1.78	0.891	1.33	0.99∼1.79	0.057
Cerebrovascular disease	1.74	1.35∼2.24	**<0.001**	1.77	1.52∼2.07	**<0.001**
Coronary artery disease	1.23	0.95∼1.60	0.121	1.35	1.15∼1.57	**<0.001**
Residence location						
Northern	1	–	–	1	–	–
Middle	0.91	0.67∼1.25	0.568	1.21	1.01∼1.44	**0.039**
Southern	1.41	1.12∼1.77	**0.004**	1.51	1.32∼1.74	**<0.001**
Eastern	1.72	1.12∼2.64	**0.014**	1.19	0.88∼1.60	0.260
Level of urbanization						
1 (most urban)	1	–	–	1	–	–
2	0.89	0.69∼1.13	0.333	0.97	0.83∼1.12	0.641
3	0.88	0.64∼1.19	0.394	0.79	0.65∼0.95	**0.014**
4	0.84	0.60∼1.17	0.291	0.85	0.70∼1.04	0.112
5 (most rural)	0.67	0.45∼1.01	0.055	0.75	0.59∼0.96	**0.020**
Income-related insured amount						
<20,000NTD/month	1	–	–	1	–	–
20,000∼39,999NTD/month	0.81	0.66∼1.01	0.059	0.86	0.76∼0.98	**0.026**
≥40,000NTD/month	0.63	0.42∼0.94	**0.023**	0.84	0.67∼1.05	0.841

Major depression: 296.2X and 296.3X.

Any depressive disorder: 296.2X, 296.3X, 300.4, and 311.

HR: hazard ratio; 95% CI: 95% confidence interval.

## Discussion

The role of the symptomatic menopausal transition and associated phenomena in the occurrence of depressive disorders has been discussed and debated for a long time. Our results supported the study hypothesis that symptomatic menopausal transition is associated with an increased risk of developing major depression or any depressive disorder in later life, independent of age, residence location, level of urbanization, monthly income, and medical comorbidities.

Several epidemiological studies have shown that the risk of mood disturbance, such as depressed mood, anxiety, and irritability, was higher in the menopausal transition, and the risk of depressive symptoms remained elevated later [3]–[4], [6], [30]–[31]. Bromberger et al followed 3302 midlife women for 8 years and demonstrated that 23% of the enrolled women had elevated Center for Epidemiologic Studies Depression (CES-D) scores, and that they were more likely to report higher CES-D scores when they were in the menopausal transition than when they were premenopausal (OR range 1.30 to 1.71) [31]. In the Penn Ovarian Aging Study (POAS), Freeman et al enrolled 231 women who had no history of depression, and found that high CES-D scores (≥16) were more than 4 times more likely to occur during a woman's menopausal transition than when she was premenopausal (OR: 4.29; 95% CI: 2.39–7.72) [32]. Beyond depressive symptoms and related psychiatric symptoms, women in the menopausal transition had an elevated likelihood of developing a new-onset depressive disorder or recurrent depressive episode [6], [10], [30], [32]. Using the data set of the SWAN study investigating the risk factors for developing the first lifetime episodes of major depression in midlife women, Bromberger et al demonstrated that during a 7-year follow-up, 42 (15.8%) women met the criteria for a diagnosis of major depression, and that frequent vasomotor symptoms (HR 2.14, 95% CI: 1.07∼4.30) and a lifetime history of any anxiety disorder were significant predictors of new-onset major depression [7]. Similar results on the association of menopausal transition with depression were noted in a recent Taiwanese study [9]. Using the Taiwanese Depression Questionnaire, and self-reported information of menopausal status and frequency of menstrual periods in the preceding 12 months, Lin et al assessed 3359 women aged 40–55 years, and demonstrated that the increase in depression was significantly associated with menopausal transition (OR: 1.97, 95%CI: 1.24–3.14), independent of menopausal symptoms [9]. Furthermore, Bromberger et al reported that women who were during the menopausal transition had a significantly increased risk of developing a major depressive episode relative to those who were at the premenopausal sate, and the effect of the menopausal state was independent of history of major depression, annually measured upsetting life events, vasomotor symptoms, and serum levels of or changes in reproductive hormones [10]. However, some previous studies did not find an association of menopausal transition with depressive disorder [11], [33]–[34]. Avis et al’s 5-year longitudinal study examined the effect of change in menopause status on depression among 2565 women aged 45 to 55 years, and demonstrated that natural menopausal transition was not associated with increased risk of depression [33]. Freeman et al followed 332 women aged 35 to 47 years for 4 years, and showed that both early menopausal transition (OR: 1.12, 95%CI: 0.75∼1.67) and late menopausal transition (OR: 0.33, 95%CI: 0.08∼1.44) were not associated with the development of major depression [11]. Vesco et al’s systemic review exhibited that there was no demonstrated pattern of an adverse independent influence of the menopausal transition on mood symptoms in mid-life women [34]. In our study, the results showed that the risks of major depression and any depressive disorder increased in those who suffered from symptomatic menopausal transition. Further clinical studies would be required to clarify this inconsistent phenomenon.

A potential pathophysiology linking hormone, menopausal stage, and depressive disorder has been proposed. The hypothesis was that the irregular pattern and the gradual decline of female gonadal hormone production during the menopausal transition increased the vulnerability to depression in susceptible women [35]–[36]. The Melbourne Women's Midlife Health Project showed that depressive symptoms were not significantly associated with the absolute serum levels of female gonadal hormones (i.e., estradiol, free estradiol, and FSH), but were significantly associated with the relative changes in hormone (i.e., a decline in total serum estradiol and a large increase in FSH level) [37]. However, both the SWAN study and the POAS study failed to validate this association. Bromberger et al’s results did not support the association between either the serum level of estradiol or the change in that level and the severity of depressive symptoms [38]; Morrison et al failed to find any association of depressive symptoms and the diagnosis of major depression with the estradiol level [39]. In addition to female gonadal hormones, some studies proposed the possible association of depressive symptoms and depressive disorder with male gonadal hormone (i.e., testosterone and dehydroepiandrosterone sulfate [DHEA-S]), but the results were inconsistent [39]–[41]. Morrison et al reported that the association between the serum DHEA-S level and depressive symptoms during the menopausal transition varied with age: in younger women, depressive symptoms were positively associated with the DHEA-S level, but in older women, they were negatively associated [12]. Bromberger et al failed to validate either a positive or negative association between the DHEA-S level and depressive symptoms, but found the log-transformed testosterone level was significantly positively associated with depressive symptoms [38]. However, Santoro et al found a negative association between the log-transformed testosterone level and depressive symptoms [40]. As can be seen, evidence of an association between gonadal hormone and depression remains inconsistent, so further studies are required to investigate the underlying pathophysiology between menopausal transition and depressive disorder.

The reciprocal relationship among menopausal transition, medical comorbidity, and depression has rarely been investigated before. Most previous studies have focused on only two of them specifically, but medical comorbidities are associated with both an increased risk of menopausal transition [15], [20], [42]–[43], and an increased risk of developing depression [14], [20], [22], [42], [44]–[46]. For example, chronic liver disease can compromise ovary function, and early menopause was noted in women with chronic hepatitis C that poorly responsed to antiviral therapy [16], [43]. Depression is often found in patients with chronic liver disease [45]–[46]. Weinstein et al reported that individuals with non-alcoholic fatty liver disease and hepatitis C virus have a higher prevalence of depression (27.2% and 29.8%, respectively) than hepatitis B virus carriers (3.7%), and the most consistent correlates of depression status in chronic liver disease patients were being female and excessive alcohol consumption [46]. Current evidence has shown that menopausal transition elevated cerebrovascular and cardiovascular risks [15], [20], which have been regarded as significant risk factors for depression [14], [24]. Post-stroke depression and post-myocardial infarction depression are commonly seen in clinical practice [14], [24]. On the contrary, Kok et al found that the risks of heart disease and atherosclerosis determined age at menopause and accelerated menopause [47]. In our study, we found that women who suffered from symptomatic menopausal transition had a higher prevalence of comorbid medical illnesses, both at enrollment and during the follow-up period, than the control group. Taking into consideration the interactive effect of these three conditions, we also assessed the effects of medical comorbidities on the risk of major depression or any depressive disorder in women with symptomatic menopausal transition in the cohort regression model. As we expected, several medical comorbidities, such as cerebrovascular diseases, coronary artery diseases, heart failure, and liver diseases, increased the risk of developing depressive disorder, but symptomatic menopausal transition was still an independent risk factor for developing depression, after adjusting medical comorbidities and related demographic characteristics. Further study would be required to investigate the underlying mechanism of the association among menopause, medical comorbidities, and depression.

Evidence regarding the association between age at menopause and the risk of developing depression is limited. Woods et al followed 302 mid-life women between 1990 and 2005, and reported that age was modestly and negatively related to depressive symptoms and CES-D scores [48]. The hypothesis was that a longer reproductive life would be related to a lower risk of depression. Indirect evidence came from the SWAN study, which revealed that a longer duration of estradiol exposure prior to the menopausal transition was linked to a lower risk of having depression [49]. In our study, those who were older at enrollment exhibited a decreased risk of developing any depressive disorder but not major depression. However, the role and underlying mechanism of age at menopause on the risk of depression is still uncertain, and further studies are needed to clarify this epidemiological phenomenon.

With regard to the effect of socioeconomic factors on depression risk, living in an urban area and lower monthly income were associated with an elevated risk of depressive disorder in our study, consistent with previous findings [50]–[51]. Wise et al’s prospective cohort study reported that incidence of menopausal transition was 1.75 times higher (95%CI: 1.10–2.79) and median age at onset was 1.2 years younger (44.7 vs. 45.9 years) for women reporting economic distress compared with women reporting no economic distress [52]. Graziottin et al suggested that in midlife women menopausal transition may act as a predisposing factor to depressive disorder when it increased vulnerability to environmental stresses (i.e., low socioeconomic status or stressful life events); as a precipitating factor, when it triggered the expression of a genetic vulnerability to major depression; and as a maintenance factor, when it gradually worsened neuronal vulnerability to both genetic and environmental factors [53]. The results implied that medical treatment alone for depressive disorder that occurs during menopausal transition was probably not enough, and that a psychosocial approach would be required for a better therapeutic outcome.

Some limitations in our study should be addressed here. First, the diagnoses of major depression and any depressive disorder may be underestimated because only those who sought medical help were enrolled in our study. But the diagnoses herein were more validated than self-reported diagnoses, because they were made by board-certificated psychiatrists. Second, symptomatic menopausal transition indeed included various somatic and psychiatric symptoms, but in our database we cannot identify the symptoms on which gynecologists based their diagnoses. Further clinical study would be required to investigate the association between the different menopausal symptoms and the development of depressive disorder. Third, some information was not supplied by NHIRD, such as personal lifestyle, stressful life events, environmental factors, family history, disease severity, menopausal stage, various menopausal symptoms, and age at onset of menopause. Those factors may be associated with the development of depressive disorder, but we could not evaluate their impact in our study.

### Conclusion

Our results supported the association between symptomatic menopausal transition and the development of depressive disorder in midlife women. Women with symptomatic menopausal transition had higher prevalence of medical comorbidities, including cerebrovascular diseases, cardiovascular diseases, and liver diseases, and exhibited an increased likelihood of developing new-onset major depression or any depressive disorder in later life. Furthermore, the symptomatic menopausal transition was significantly associated with an elevated risk of major depression and any depressive disorder independent of the aforementioned medical comorbidities. Further clinical studies would be required to validate our results and explore whether the prompt intervention of symptomatic menopausal transition may decrease the risk of developing depressive disorder in later life.
